# Supplementary material for: Climate change impacts on ocean light in Arctic ecosystems
Source: Nat Commun. 2025 Nov 6;16:9798. doi: 10.1038/s41467-025-64790-4 (PMC12592436; doi:10.1038/s41467-025-64790-4)
Supplement: Supplementary file 1 — Supplementary Information [file 41467_2025_64790_MOESM1_ESM.pdf]

Supplementary information to:

Climate change impacts on ocean light in Arctic ecosystems

Trond Kristiansen[1,2], Øystein Varpe[3,4], Elizabeth R. Selig[5], Benjamin J. Laurel[6], William J. Sydeman[1], Michaela I. Hegglin[7,8], and Phil Wallhead[9]

1* Farallon Institute, Petaluma, USA

2 Actea Inc, San Francisco, California, USA

3 Department of Biological Sciences, University of Bergen, and Bjerknes Centre for Climate Research, Bergen, Norway

4 Norwegian Institute for Nature Research, Bergen, Norway

5 Stanford Center for Ocean Solutions, Stanford University, Stanford, USA

6 Alaska Fisheries Science Center, NOAA, Newport, USA

7 Institute of Climate and Energy Systems – Stratospere (ICE-4), Forschungszentrum Jülich, Julich, Germany

8 Department of Meteorology, University of Reading, Reading, UK

9 Norwegian Institute for Water Research, Oslo, Norway

***email corresponding author: trondkr@faralloninstitute.org**

CMIP6 model	Institution	Experiment ID	Member ID
CanESM5	The Canadian Earth System Model version 5[1]	SSP2-4.5<br>SSP5-8.5	r1i1p2f1<br>r2i1p1f1<br>r3i1p2f1<br>r7i1p2f1<br>r9i1p2f1<br>r10i1p2f1
MPI-ESM1-2-LR	Max Planck Institute Earth System Model Low-res[2,3]	SSP2-4.5<br>SSP5-8.5	r1i1p1f1<br>r2i1p1f1<br>r6i1p1f1<br>r10i1p1f1
MPI-ESM1-2-HR	Max Planck Institute Earth System Model High-res[2,3]	SSP2-4.5<br>SSP5-8.5	r1i1p1f1<br>r2i1p1f1
UKESM1-0-LL	U.K. Community Earth System Modeling for CMIP6[4,5]	SSP2-4.5<br>SSP5-8.5	r1i1p1f2<br>r2i1p1f2<br>r3i1p1f2<br>r4i1p1f2

Supplementary Table 1: An overview of the CMIP6 models, experiment, and ensemble members used to calculate the shortwave light calculations in the ocean. For each model, a range (2-6) of model realizations (member ids) were used for each of the two climate experiments SSP2-4.5 and SSP5-8.5.

Model-realization-scenario	LME	r	RMSE (W/m2)	albedo
CanESM5_r3i1p2f1_ssp245	Barents Sea	0.965	2.786	0.179
CanESM5_r3i1p2f1_ssp245	Northern Bering - Chukchi Seas	0.972	4.365	0.396
CanESM5_r7i1p2f1_ssp245	Barents Sea	0.965	3.011	0.196
CanESM5_r7i1p2f1_ssp245	Northern Bering - Chukchi Seas	0.973	4.63	0.387
CanESM5_r10i1p2f1_ssp245	Barents Sea	0.947	3.142	0.188
CanESM5_r10i1p2f1_ssp245	Northern Bering - Chukchi Seas	0.981	4.559	0.382
MPI-ESM1-2-HR_r1i1p1f1_ssp245	Barents Sea	0.951	2.461	0.27
MPI-ESM1-2-HR_r1i1p1f1_ssp245	Northern Bering - Chukchi Seas	0.832	2.017	0.389
MPI-ESM1-2-HR_r2i1p1f1_ssp245	Barents Sea	0.934	2.87	0.248
MPI-ESM1-2-HR_r2i1p1f1_ssp245	Northern Bering - Chukchi Seas	0.786	2.295	0.38
MPI-ESM1-2-LR_r1i1p1f1_ssp245	Barents Sea	0.913	5.555	0.219
MPI-ESM1-2-LR_r1i1p1f1_ssp245	Northern Bering - Chukchi Seas	0.796	5.453	0.379
MPI-ESM1-2-LR_r2i1p1f1_ssp245	Barents Sea	0.91	5.174	0.222
MPI-ESM1-2-LR_r2i1p1f1_ssp245	Northern Bering - Chukchi Seas	0.757	5.694	0.38
CanESM5_r9i1p2f1_ssp245	Barents Sea	0.957	2.919	0.185
CanESM5_r9i1p2f1_ssp245	Northern Bering - Chukchi Seas	0.974	4.588	0.388
CanESM5_r1i1p2f1_ssp245	Barents Sea	0.956	3.146	0.191
CanESM5_r1i1p2f1_ssp245	Northern Bering - Chukchi Seas	0.971	4.775	0.391
CanESM5_r2i1p2f1_ssp245	Barents Sea	0.935	3.374	0.186
CanESM5_r2i1p2f1_ssp245	Northern Bering - Chukchi Seas	0.973	4.619	0.387
MPI-ESM1-2-LR_r6i1p1f1_ssp245	Barents Sea	0.924	5.297	0.224
MPI-ESM1-2-LR_r6i1p1f1_ssp245	Northern Bering - Chukchi Seas	0.777	5.613	0.383
MPI-ESM1-2-LR_r10i1p1f1_ssp245	Barents Sea	0.885	5.473	0.213
MPI-ESM1-2-LR_r10i1p1f1_ssp245	Northern Bering - Chukchi Seas	0.824	5.877	0.379
UKESM1-0-LL_r2i1p1f2_ssp245	Barents Sea	0.986	7.104	0.249
UKESM1-0-LL_r2i1p1f2_ssp245	Northern Bering - Chukchi Seas	0.974	12.364	0.421
UKESM1-0-LL_r1i1p1f2_ssp245	Barents Sea	0.987	7.123	0.242
UKESM1-0-LL_r1i1p1f2_ssp245	Northern Bering - Chukchi Seas	0.964	12.37	0.422
UKESM1-0-LL_r3i1p1f2_ssp245	Barents Sea	0.981	7.556	0.248
UKESM1-0-LL_r3i1p1f2_ssp245	Northern Bering - Chukchi Seas	0.964	12.225	0.42
UKESM1-0-LL_r4i1p1f2_ssp245	Barents Sea	0.986	7.358	0.237
UKESM1-0-LL_r4i1p1f2_ssp245	Northern Bering - Chukchi Seas	0.964	12.271	0.418
**Average**	**Barents Sea**	**0.95**	**4.6**	**0.22**
**Average**	**Northern Bering - Chukchi Seas**	**0.91**	**6.5**	**0.40**

Supplementary Table 2: Temporal correlation (r) and RMSE of shortwave radiation as derived by the RTM compared to the CMIP6 model output averaged over LMEs the Barents and the Northern Bering and Chukchi Seas. Calculations were done for the SSP2-4.5 scenario for each CMIP6 model and scenario considered in this study. Also shown is the average albedo averaged across each LME for the period 1979-2100 to highlight differences in

surface properties across models and realizations.

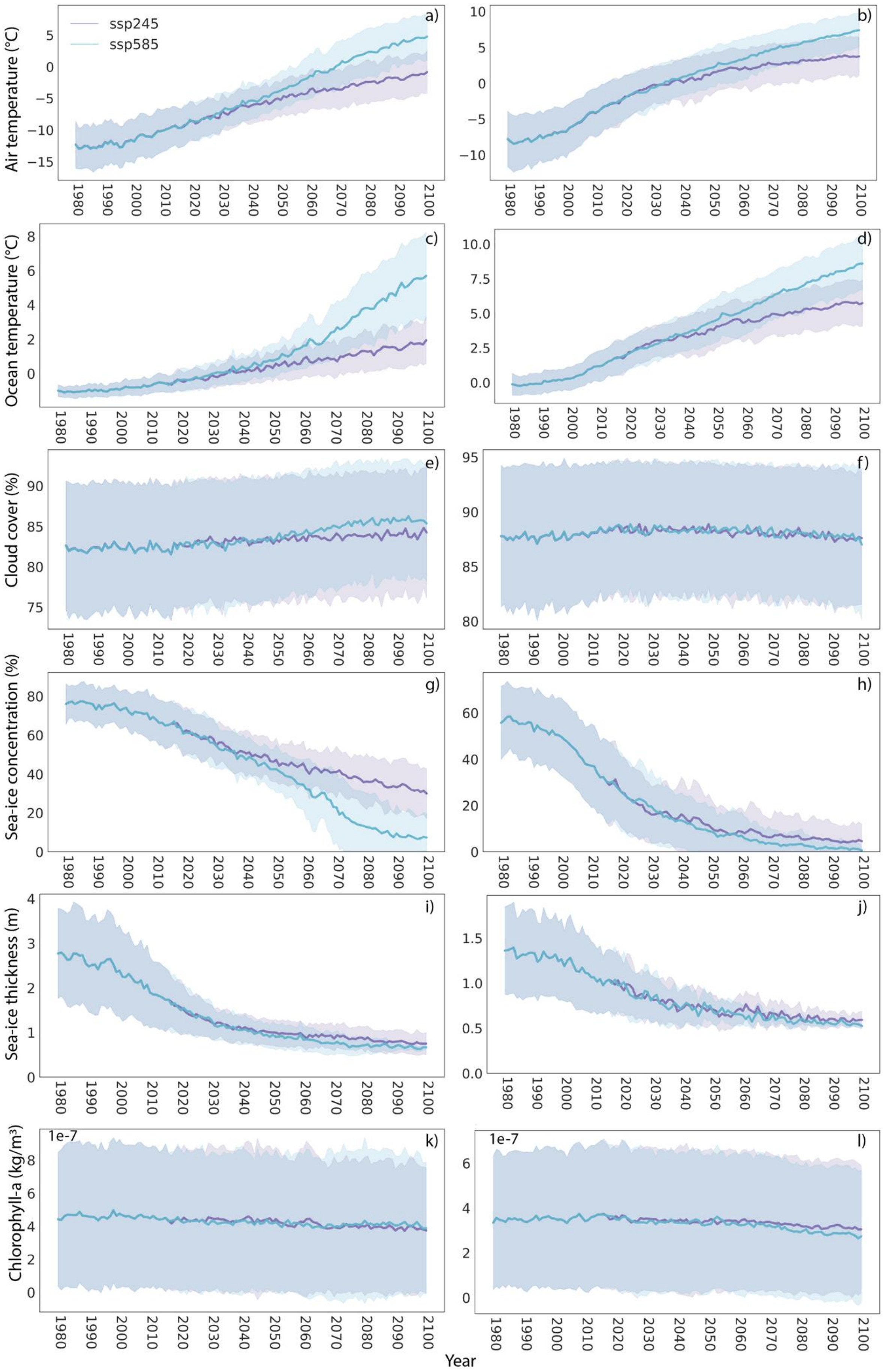
ssp245
ssp585
Air temperature (°C)
Ocean temperature (°C)
Cloud cover (%)
Sea-ice concentration (%)
Sea-ice thickness (m)
Chlorophyll-a (kg/m³)
1e-7
a)
b)
c)
d)
e)
f)
g)
h)
i)
j)
k)
l)
Year

Supplementary Figure 1: Large-scale changes in physics and biology part 1. Annual ensemble average shown with standard deviation intervals across CMIP6 models/realizations (Table 1) area averaged over the Northern Bering and Chukchi LME (left), and the Barents Sea LME (right). The panels show projected values of a, b) air temperature (ºC), c, d) ocean surface temperature (ºC), e, f) cloud cover (%), g, h) sea-ice concentration (%), i, j) sea ice thickness (m), k, l), and chlorophyll (kg/m^3) used as input to the radiative transfer model.

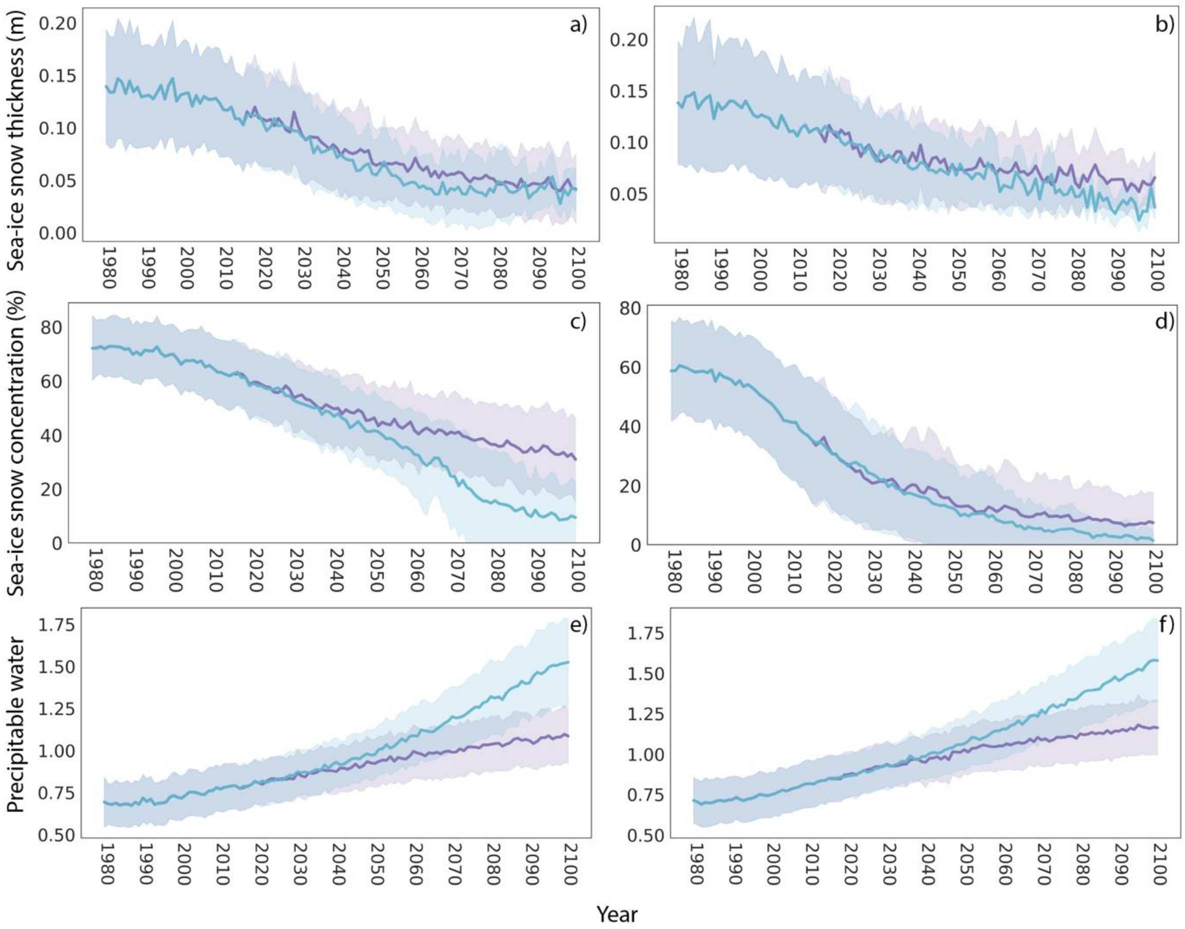

Supplementary Figure 2: Large-scale changes in physics and biology part 2. Annual ensemble average shown with standard deviation intervals across CMIP6 models/realizations (Table 1) area averaged over the Northern Bering and Chukchi LME (left), and the Barents Sea LME (right). The panels show projected values of a, b) sea-ice snow thickness (m), c, d) sea-ice snow concentration (%), e, f) precipitable water, the total amount of water vapor present in a vertical column of the atmosphere, used as input to the radiative transfer model.

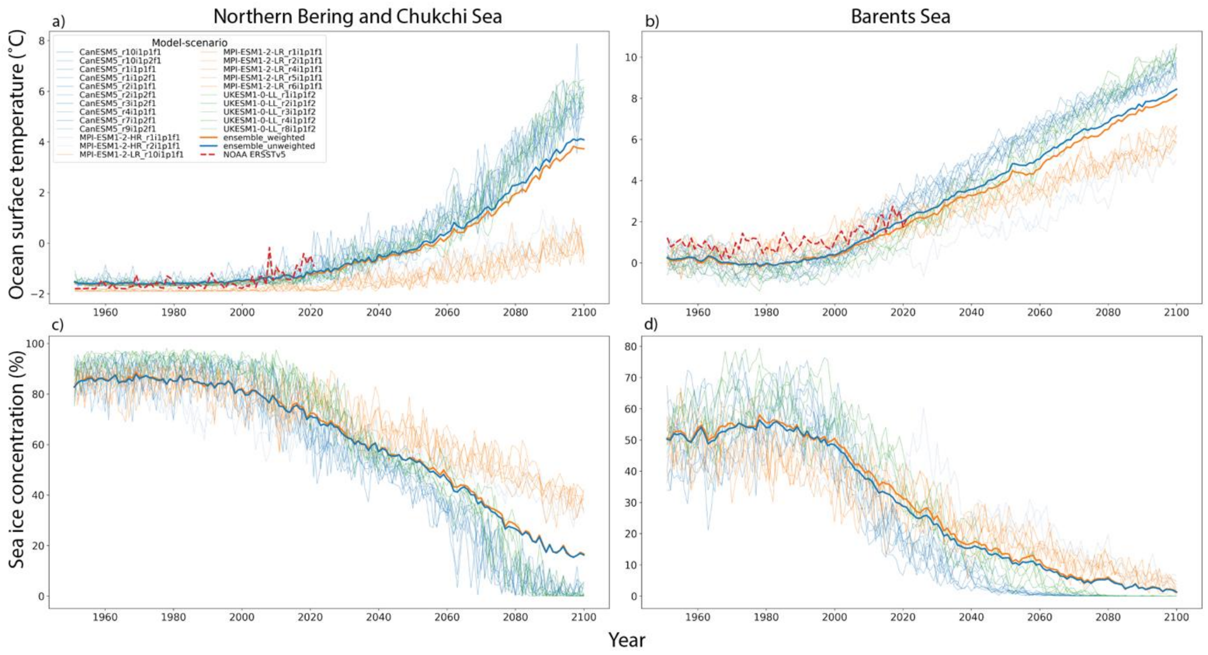

Supplementary Figure 3*: Projected changes in ocean surface temperature and sea ice concentration.* Expected changes in ocean surface temperature (ºC) and sea ice concentration (%) for (a, c) the Northern Bering and Chukchi Sea and (b, d) the Barents Sea large marine ecosystems for climate change scenario SSP5-8.5. Projections are shown for each of the individual CMIP6 model and realization combinations used as forcing for the RTM. Thick blue and orange lines show the ensemble average without and with weighting based on skill and independence. Red dashed line (a, b) shows observed sea surface temperature from NOAA ERSSTv5 dataset.

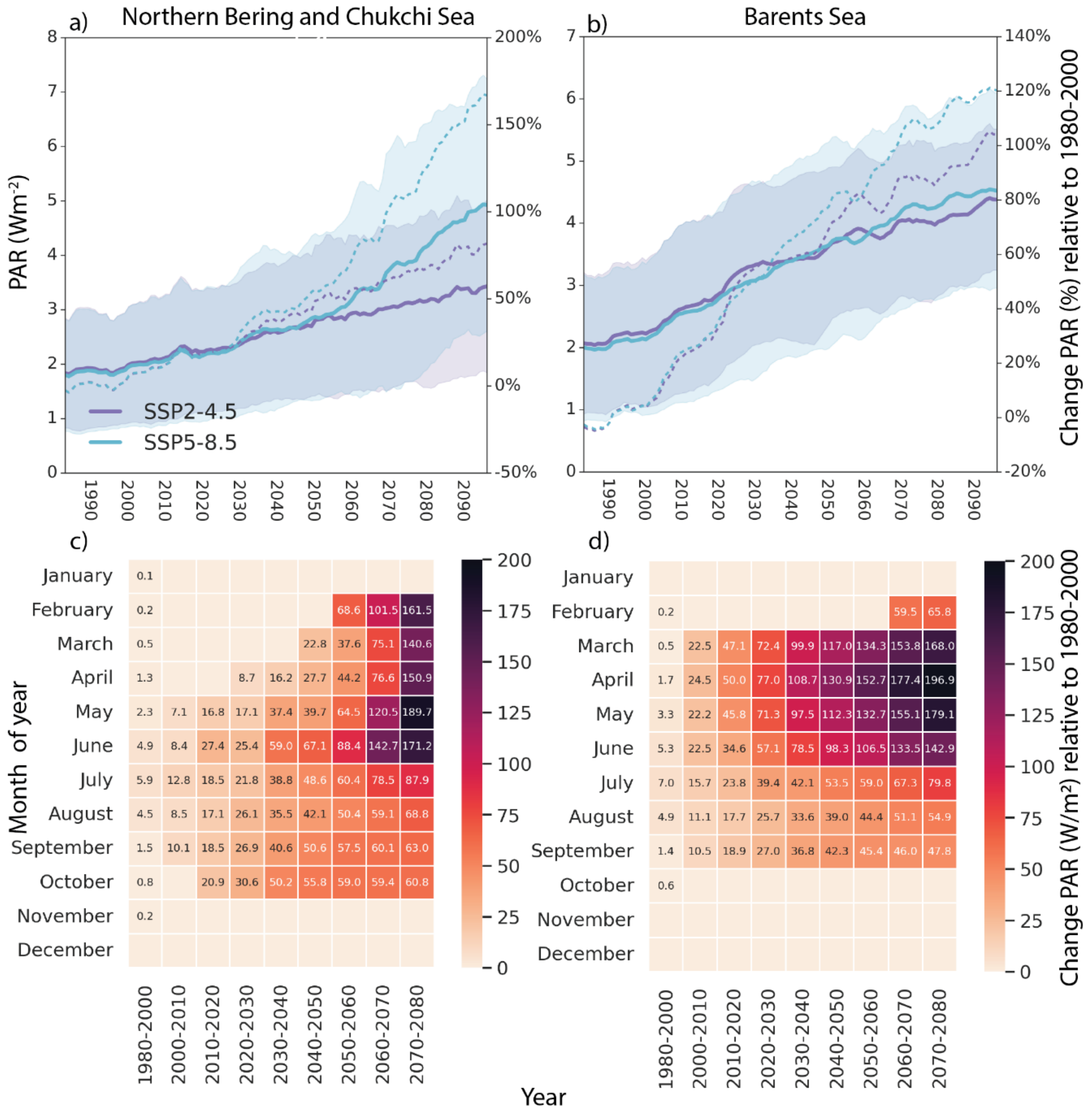

Supplementary Figure 4: Annual and seasonal changes in PAR. Future projected annual area average photosynthetic active radiation (PAR, Wm^{-2}) for a) Northern Bering and Chukchi Sea and b) the Barents Sea under climate scenarios SSP2-4.5 and SSP5-8.5. Heatmaps showing the historical (1980-2000) monthly mean (leftmost column of each heatmap) and future decadal average changes in PAR (Wm^{-2}) for the Northern Bering and Chukchi (c) and the Barents Seas (d). Changes relative to historical values (1980-2000) are shown for each decade between 2000-2090 at 10-year intervals. Months with no change relative to historical values have no annotated values. Heatmap depicts only results for SSP5-8.5.

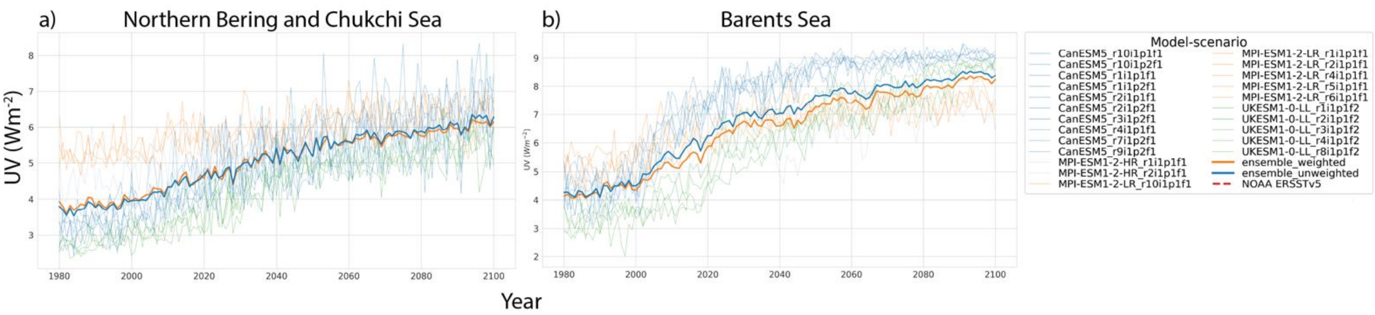

Supplementary Figure 5: Temporal changes in UV. Upper panels: Expected changes in UV (Wm^{-2}) light reaching the top of the water column in the ocean for a) the Northern Bering and Chukchi Sea, and b) the Barents Sea LME for climate change scenario SSP2-4.5. Projections are shown for each of the individual CMIP6 model and realization combinations used as forcing for the RTM. Thick blue and orange lines show the ensemble average with and without weighting based on model skill and independence.

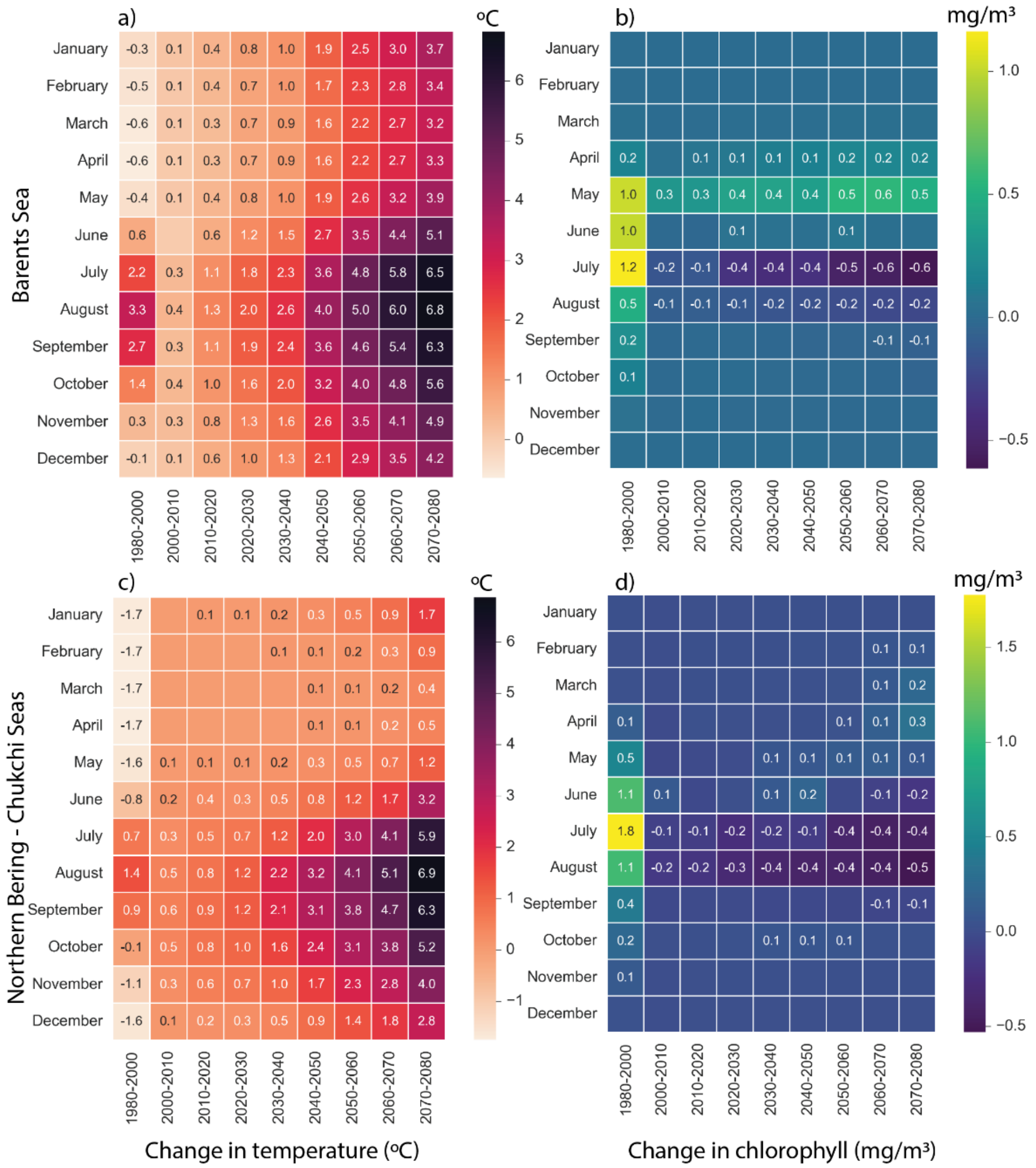

Supplementary Figure 6: Expected changes in temperature and chlorophyll abundance under SSP5-8.5. Heatmaps show the historical (1980-2000) monthly mean (leftmost column of each heatmap) and future decadal average changes in temperature (°C) in a) the Barents Sea and b) Northern Bering and Chukchi Sea and chlorophyll (mgm^{-3}) for c) the Barents and d) Northern Bering/Chukchi Seas under SSP5-8.5. Changes relative to historical values (1980-2000) are shown for each decade between 2000-2090 at 10-year intervals. Months with no change relative to historical values have no annotated value.

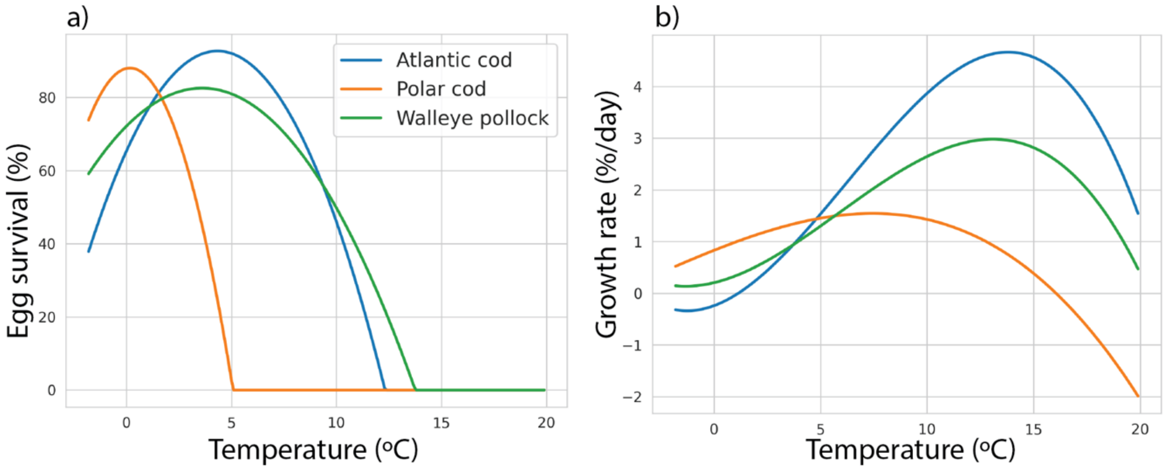

Supplementary Figure 7: Theoretical models for egg survival and juvenile growth rates under varying temperature. Functions are shown for a) egg survival (%) and b) growth rates (%day^{-1}) for Atlantic cod, Polar cod, and Walleye pollock and their response and optima in relation to ocean temperature[6–8].

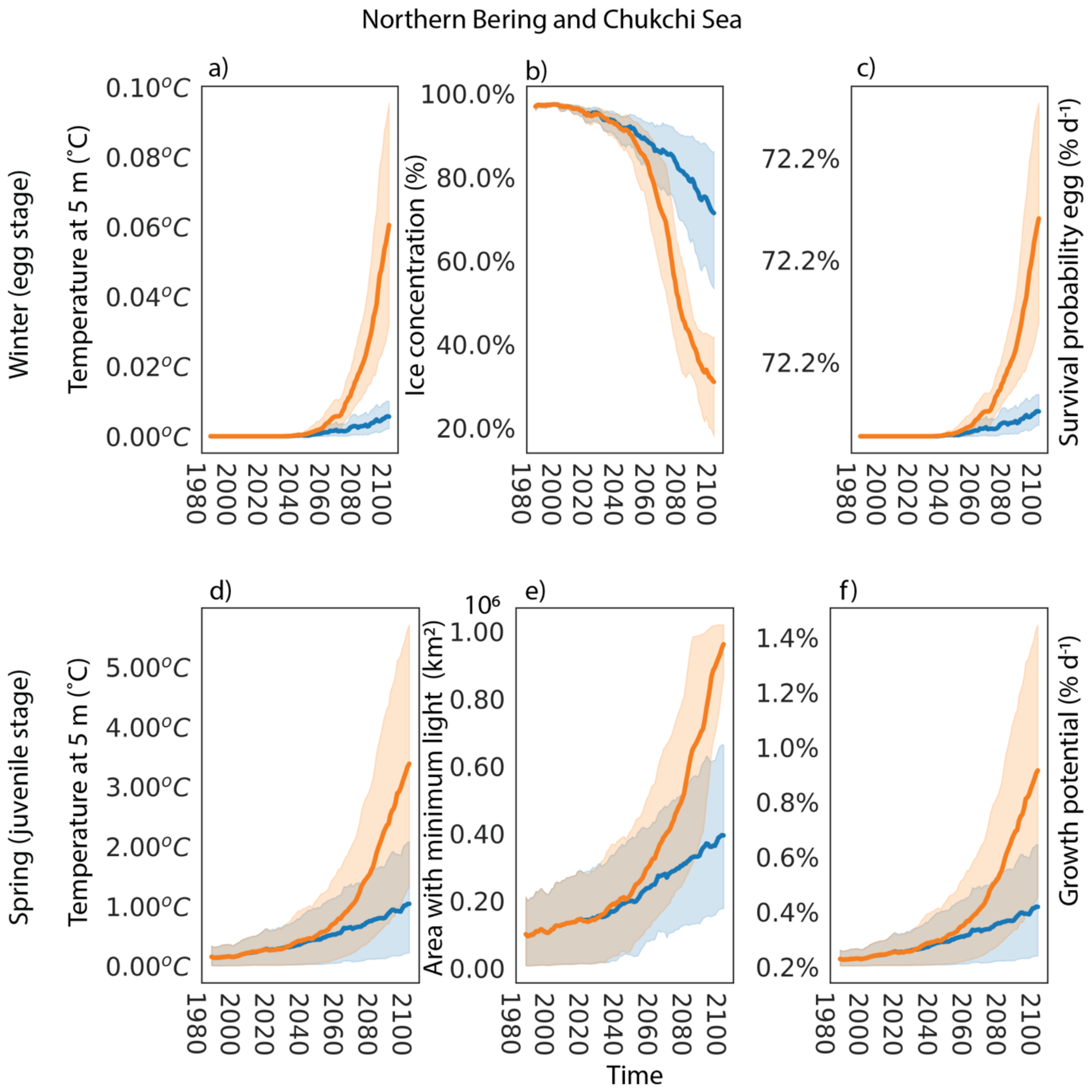

Supplementary Figure 8: Impacts of temperature and light on the egg survival and larval growth potential for Walleye pollock in the Northern Bering/Chukchi Sea. Conditions during winter months (Jan-Mar) for the egg stage are shown for a) ocean temperature (ºC) at 5 m depth, b) ice concentration, c) egg survival (% d^{-1}), while conditions during summer months for juveniles are shown for d) ocean temperature (ºC) at 5 m depth, e) area (km^2) within the LME with minimum light threshold, and f) juvenile growth potential (% d^{-1}). Thick lines show the average values across all winter/spring months for each scenario SSP2-4.5 (blue) and SSP5-8.5 (orange) while shaded regions show the 95th percentile within the winter/spring months. Survival during the egg stage and growth potential was calculated using functional relationships from laboratory studies and observations.

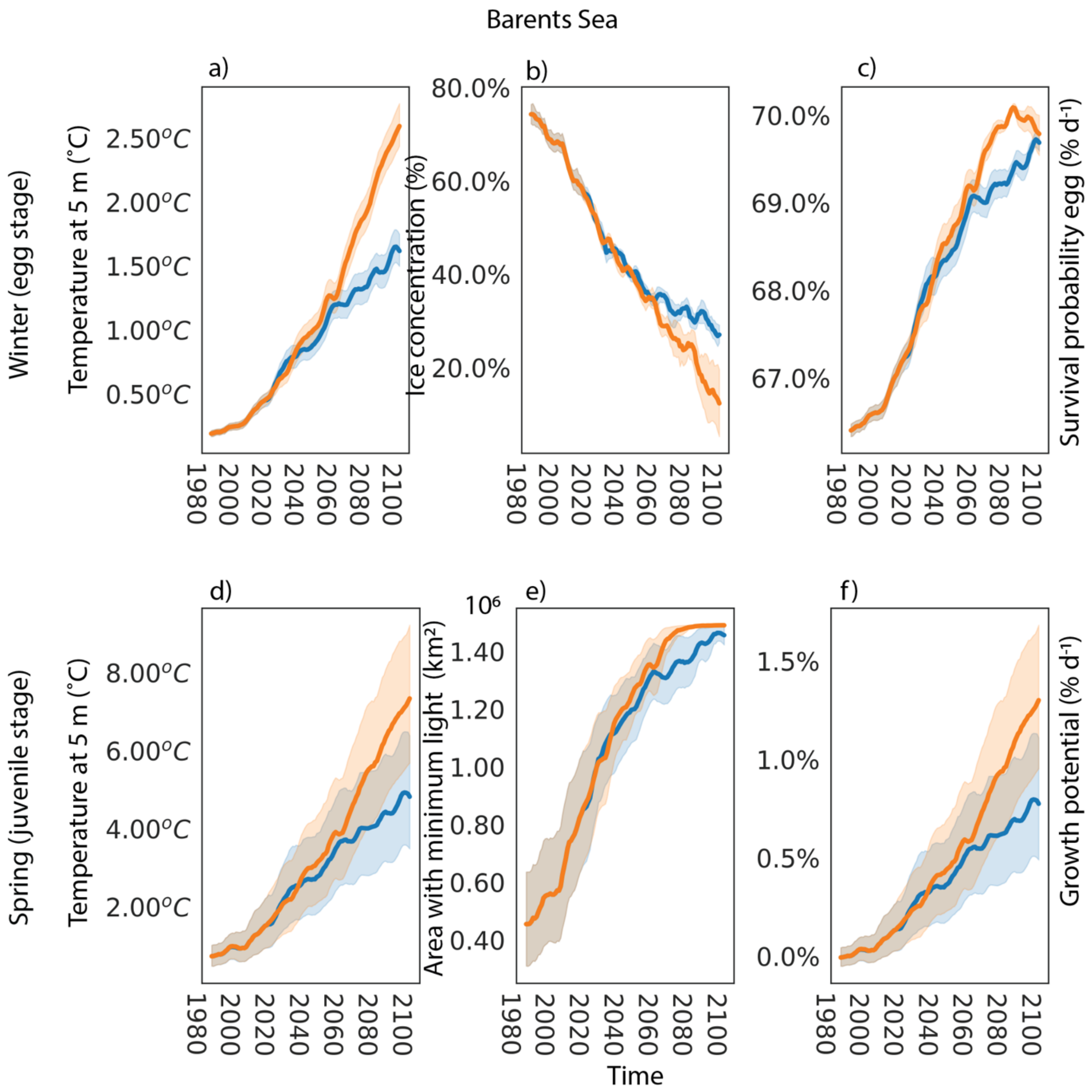

Supplementary Figure 9: Impacts of temperature and light on the egg survival and larval growth potential for Atlantic cod in Barents Sea. Northern Bering and Chukchi Sea (upper) and Barents Sea (lower) panels for winter months (Jan-Mar) and the egg stage are shown in a) ocean temperature (ºC) at 5 m depth, b) ice concentration, c) egg survival (% d^{-1}), while summer months for juveniles shown in d) ocean temperature (ºC) at 5 m depth, e) area (km^2) within the LME with minimum light threshold, and f) juvenile growth potential (% d^{-1}). Thick lines show the average values across all winter/spring months for each scenario SSP2-4.5 (blue) and SSP5-8.5 (orange) while shaded regions show the 95th percentile within the winter/spring months. Survival during the egg stage and growth potential was calculated using functional relationships from laboratory studies and observations.

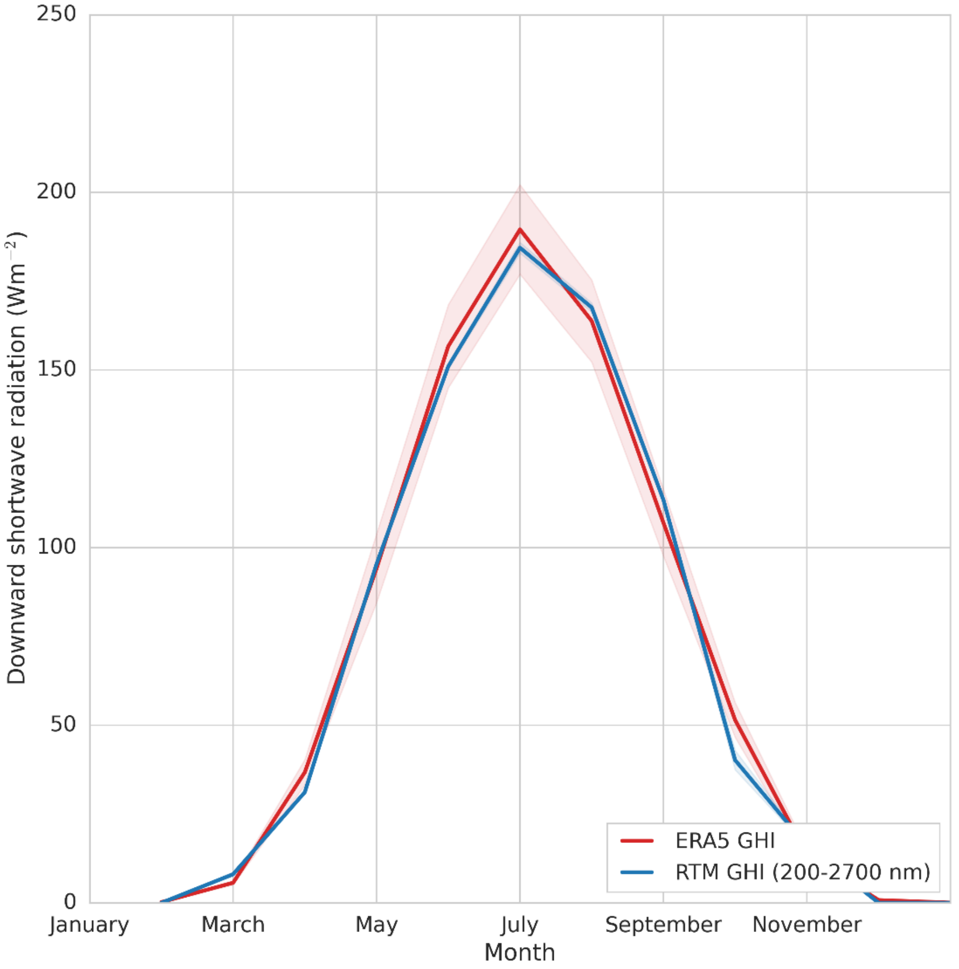

Supplementary Figure 10: Comparison ERA5 and RTM calculated shortwave radiation Barents Sea. Incoming shortwave radiation (GHI, 200-2700 nm) calculated by the RTM forced with historical CMIP6 model results compared with ERA5 mean downward shortwave radiation at the surface (**msdwswsrf**) averaged for the Barents Sea LME for the period 1979-2020, after bias-correcting the RTM with a constant factor. Shaded areas indicate the standard deviation.

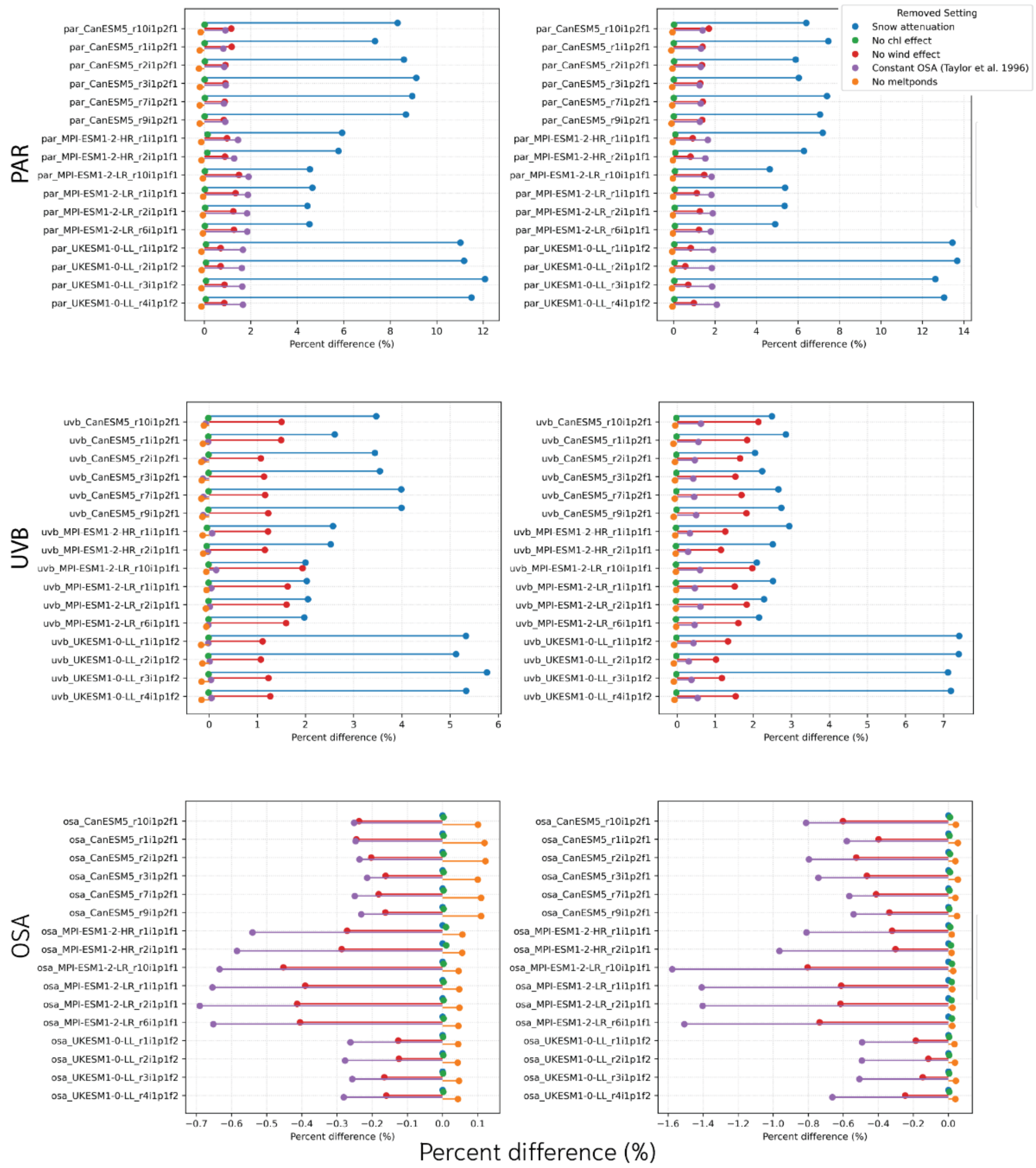

Supplementary Figure 11: Sensitivity tests of various components of the RTM and their effect on key output variables (PAR, UVB) and component of the RTM (OSA = Ocean Surface Albedo). The annual averaged percent difference to the standard RTM setup demonstrates the effect of the change. The RTM was run for 10 years (1/1/1979 to 1/1/1989) with either a feature turned on or off, or a change in parameter. Features that were turned on or off included: the effect of melt ponds (changes in albedo), wind (changes in surface roughness and albedo), chlorophyll (changes in albedo and attenuation), sea ice (changes in attenuation and albedo, but not shown as the effect makes it difficult to use the same scale), OSA (when turned off we used 0.06 as default ocean albedo), and effect of using snow attenuation coefficient of 5.9 m^{-1} versus the default 20 m^{-1}. The percent impact each feature had on PAR (upper panel), UVB (middle panel), and OSA (lower panel) is shown for the

Northern Bering/Chukchi Sea (left column) and the Barents Sea (right column). For OSA, all the features except removing melt ponds, had a negative effect on albedo (smaller value).

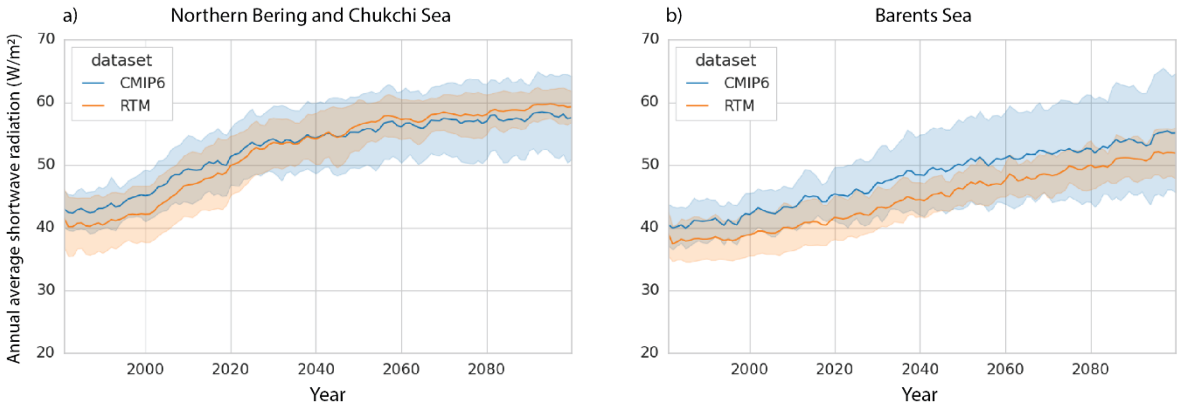

Supplementary Figure 12: Comparison between CMIP6 and the RTM model for shortwave radiation between 1979-2100 for a) Northern Bering and Chukchi Sea and b) the Barents Sea. Overall correlation was r=0.91 and RMSE=6.5 Wm^{-2} for the Northern Bering and Chukchi Sea and r=0.95 and RMSE=4.6 Wm^{-2} for the Barents Sea.

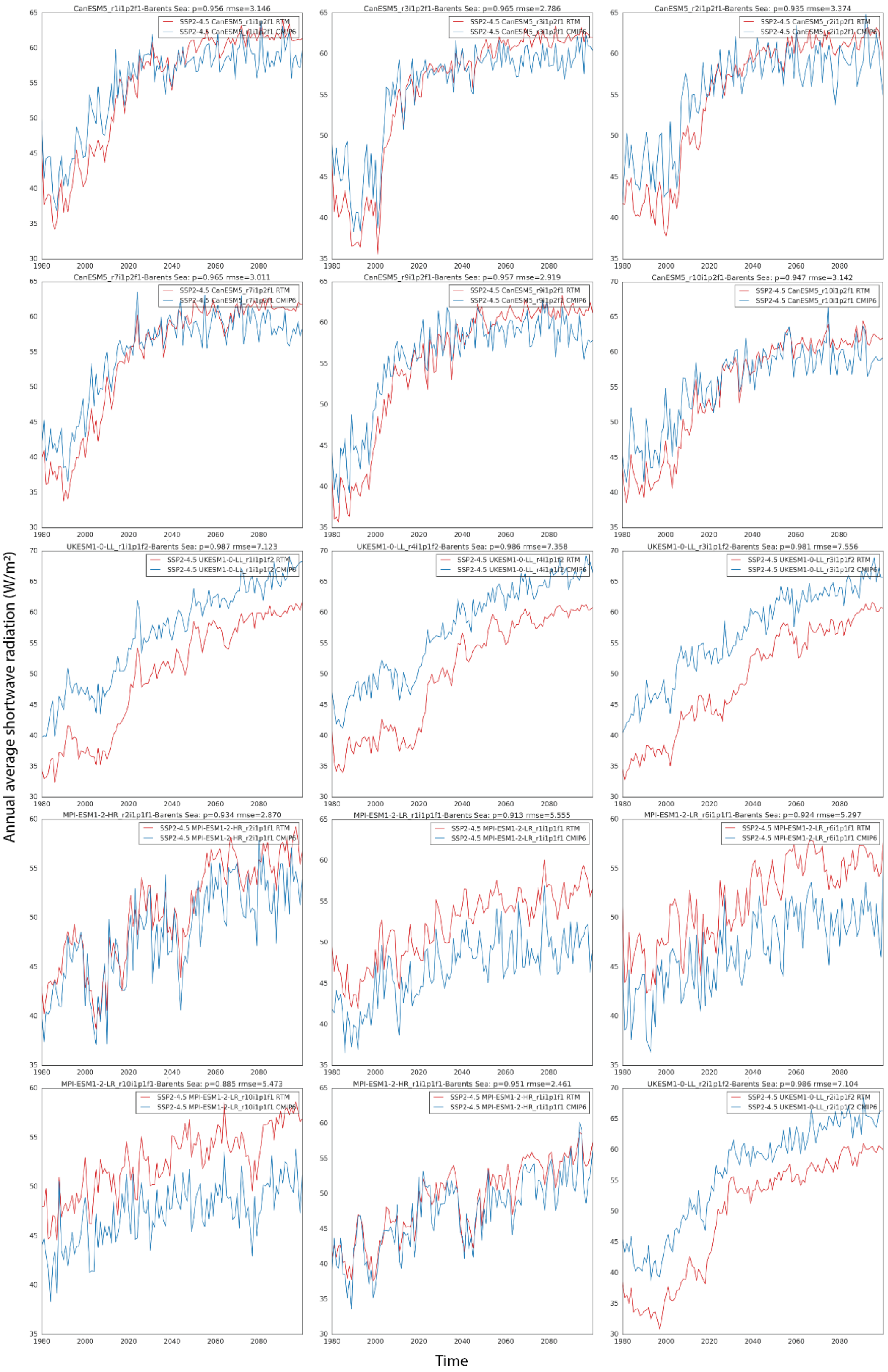

Supplementary Figure 13: Comparison between individual CMIP6 and the RTM models for shortwave radiation between 1979-2100 for the Barents Sea. The individual RMSE and temporal correlations between RTM and CMIP6 models are listed in Table S2.

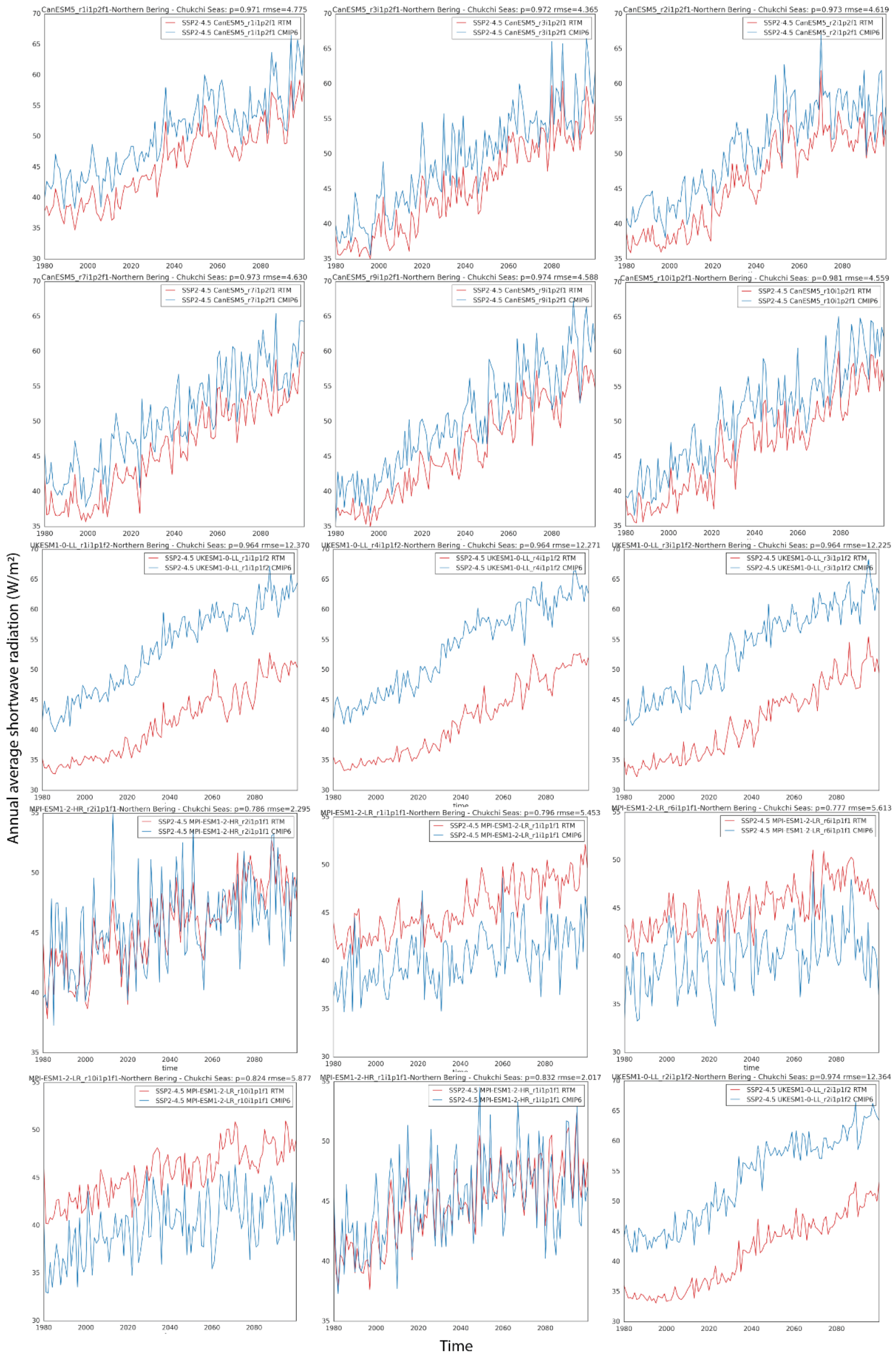

Supplementary Figure 14: Comparison between individual CMIP6 and the RTM models for shortwave radiation between 1979-2100 for the Northern Bering and Chukchi Sea. The individual RMSE and temporal correlations between RTM and CMIP6 models are listed in Table S2.

References

1. Swart, N. C. *et al.* The Canadian Earth System Model version 5 (CanESM5.0.3). *Geosci. Model Dev.* **12**, 4823–4873 (2019).
2. Mauritsen, T. *et al.* Developments in the MPI-M Earth System Model version 1.2 (MPI-ESM1.2) and Its Response to Increasing CO2. *J Adv Model Earth Syst* **11**, 998–1038 (2019).
3. Gutjahr, O. *et al.* Max Planck Institute Earth system model (MPI-ESM1.2) for the high-resolution model intercomparison project (HighResMIP). *Geosci. Model Dev.* **12**, 3241–3281 (2019).
4. Sellar, A. A. *et al.* UKESM1: Description and evaluation of the U.k. earth system model. *J. Adv. Model. Earth Syst.* **11**, 4513–4558 (2019).
5. Yool, A. *et al.* Evaluating the physical and biogeochemical state of the global ocean component of UKESM1 in CMIP6 historical simulations. *Geosci. Model Dev.* **14**, 3437–3472 (2021).
6. Laurel, B. J., Copeman, L. A., Spencer, M. & Iseri, P. Comparative effects of temperature on rates of development and survival of eggs and yolk-sac larvae of Arctic cod (Boreogadus saida) and walleye pollock (Gadus chalcogrammus). *ICES J. Mar. Sci.* **75**, 2403–2412 (2018).
7. Laurel, B. J., Spencer, M., Iseri, P. & Copeman, L. A. Temperature-dependent growth and behavior of juvenile Arctic cod (Boreogadus saida) and co-occurring North Pacific gadids. *Polar Biology* vol. 39 1127–1135 Preprint at https://doi.org/10.1007/s00300-015-1761-5 (2016).
8. Björnsson, B., Steinarsson, A. & Árnason, T. Growth model for Atlantic cod (Gadus morhua): Effects of temperature and body weight on growth rate. *Aquaculture* **271**, 216–226 (2007).
