# Supplementary material for: Climate change impacts on ocean light in Arctic ecosystems
Source: Nat Commun. 2025 Nov 6;16:9798. doi: 10.1038/s41467-025-64790-4 (PMC12592436; doi:10.1038/s41467-025-64790-4)
Supplement: Supplementary file 2 — Reporting Summary [file 41467_2025_64790_MOESM2_ESM.pdf]

nature portfolio

Corresponding author(s): Trond Kristiansen

Last updated by author(s): May 28, 2025

Reporting Summary

Nature Portfolio wishes to improve the reproducibility of the work that we publish. This form provides structure for consistency and transparency in reporting. For further information on Nature Portfolio policies, see our Editorial Policies and the Editorial Policy Checklist.

Statistics

For all statistical analyses, confirm that the following items are present in the figure legend, table legend, main text, or Methods section.

n/a	Confirmed	
☐	☒	The exact sample size (*n*) for each experimental group/condition, given as a discrete number and unit of measurement
☒	☐	A statement on whether measurements were taken from distinct samples or whether the same sample was measured repeatedly
☒	☐	The statistical test(s) used AND whether they are one- or two-sided<br>*Only common tests should be described solely by name; describe more complex techniques in the Methods section.*
☒	☐	A description of all covariates tested
☐	☒	A description of any assumptions or corrections, such as tests of normality and adjustment for multiple comparisons
☐	☒	A full description of the statistical parameters including central tendency (e.g. means) or other basic estimates (e.g. regression coefficient) AND variation (e.g. standard deviation) or associated estimates of uncertainty (e.g. confidence intervals)
☒	☐	For null hypothesis testing, the test statistic (e.g. *F*, *t*, *r*) with confidence intervals, effect sizes, degrees of freedom and *P* value noted<br>*Give P values as exact values whenever suitable.*
☒	☐	For Bayesian analysis, information on the choice of priors and Markov chain Monte Carlo settings
☒	☐	For hierarchical and complex designs, identification of the appropriate level for tests and full reporting of outcomes
☒	☐	Estimates of effect sizes (e.g. Cohen's *d*, Pearson's *r*), indicating how they were calculated

Our web collection on statistics for biologists contains articles on many of the points above.

Software and code

Policy information about availability of computer code

Data collection	Source code to run the Radiative Transfer Model is available on Github (DOI:10.5281/zenodo.15530836) and CodeOcean (https://codeocean.com/capsule/0694935/tree)
Data analysis	All data analysis were done using the RTM source code.

For manuscripts utilizing custom algorithms or software that are central to the research but not yet described in published literature, software must be made available to editors and reviewers. We strongly encourage code deposition in a community repository (e.g. GitHub). See the Nature Portfolio guidelines for submitting code & software for further information.

Data

Policy information about availability of data

All manuscripts must include a data availability statement. This statement should provide the following information, where applicable:

- Accession codes, unique identifiers, or web links for publicly available datasets
- A description of any restrictions on data availability
- For clinical datasets or third party data, please ensure that the statement adheres to our policy

The CMIP6 datasets used as input to the RTM are freely available through Google Cloud Storage (see console.cloud.google.com/storage/browser/cmip6). Code to generate the necessary forcing files to run the RTM is included in the RTM source code.

Research involving human participants, their data, or biological material

Policy information about studies with human participants or human data. See also policy information about sex, gender (identity/presentation), and sexual orientation and race, ethnicity and racism.

Reporting on sex and gender	N/A
Reporting on race, ethnicity, or other socially relevant groupings	N/A
Population characteristics	N/A
Recruitment	N/A
Ethics oversight	N/A

Note that full information on the approval of the study protocol must also be provided in the manuscript.

Field-specific reporting

Please select the one below that is the best fit for your research. If you are not sure, read the appropriate sections before making your selection.

☐ Life sciences ☐ Behavioural & social sciences ☒ Ecological, evolutionary & environmental sciences

For a reference copy of the document with all sections, see nature.com/documents/nr-reporting-summary-flat.pdf

Ecological, evolutionary & environmental sciences study design

All studies must disclose on these points even when the disclosure is negative.

Study description	This study developed a radiative transfer model (RTM) to quantify the amount of light that will reach the ocean surface waters under various climate models and climate scenarios. We use the light values to project consequences on various fish species typically found at high latitudes.
Research sample	N/A
Sampling strategy	N/A
Data collection	Data used in this study originated from global climate models developed as part of the Common Model InterComparison Project phase 6 (CMIP6). The CMIP6 datasets used as input to the RTM are freely available through Google Cloud Storage (see console.cloud.google.com/storage/browser/cmip6).
Timing and spatial scale	The timing of the data used were 1979-2100. The frequency of the data were monthly.
Data exclusions	We selected a representative subset of CMIP6 models based on their skill at simulating ocean climate conditions at high latitudes.
Reproducibility	The RTM model code available on CodeOcean as a capsule linked to this manuscript and on Gihub would reproduce our results as each input forcing file is directly created using the CMIP6 datasets available on Google Cloud Storage.
Randomization	N/A
Blinding	N/A

Did the study involve field work? ☐ Yes ☒ No

Reporting for specific materials, systems and methods

We require information from authors about some types of materials, experimental systems and methods used in many studies. Here, indicate whether each material, system or method listed is relevant to your study. If you are not sure if a list item applies to your research, read the appropriate section before selecting a response.

Materials & experimental systems

n/a	Involved in the study
☒	☐ Antibodies
☒	☐ Eukaryotic cell lines
☒	☐ Palaeontology and archaeology
☒	☐ Animals and other organisms
☒	☐ Clinical data
☒	☐ Dual use research of concern
☒	☐ Plants

Methods

n/a	Involved in the study
☒	☐ ChIP-seq
☒	☐ Flow cytometry
☒	☐ MRI-based neuroimaging

Plants

Seed stocks	N/A
Novel plant genotypes	N/A
Authentication	N/A
